# Arf6 regulates EGF-induced internalization of *E*-cadherin in breast cancer cells

**DOI:** 10.1186/s12935-015-0159-3

**Published:** 2015-02-04

**Authors:** Rui Xu, Yujie Zhang, Luo Gu, Jianchao Zheng, Jie Cui, Jing Dong, Jun Du

**Affiliations:** State Key Laboratory of Reproductive Medicine, Nanjing Medical University, 140 Hanzhong Road, Nanjing, Jiangsu 210029 China; Department of Physiology, Nanjing Medical University, Nanjing, Jiangsu 210029 China; Department of Biotechnology, Nanjing Medical University, Nanjing, Jiangsu 210029 China; Department of Biochemistry and Molecular Biology, Nanjing Medical University, Nanjing, Jiangsu 210029 China; Department of Epidemiology and Biostatistics and Ministry of Education (MOE) Key Lab for Modern Toxicology, Nanjing Medical University, Nanjing, Jiangsu 210029 China

**Keywords:** EGF, Arf6, *E*-cadherin, Internalization, Breast cancer

## Abstract

*E*-cadherin internalization facilitates dissolution of adherens junctions and promotes tumor cell epithelial-mesenchymal transition (EMT) and migration. Our previous results have shown that Arf6 exerts pro-migratory action in breast cancer cells after EGF stimulation. Despite the fact that EGF signaling stimulates EMT of breast cancer cells, the effect of Arf6 on internalization of *E*-cadherin of breast cancer cells under EGF treatment remains to be determined. Here, we showed that EGF dose-dependently stimulated *E*-cadherin internalization by MCF-7 cells with the maximal effect at 50 ng/ml. Meanwhile, EGF treatment markedly increased Arf6 activation. Arf6 was involved in complexes of *E*-cadherin, and more *E*-cadherin was pulled down with Arf6 when the activity of the latter was increased. Immunoblotting and immunofluorescence assays showed that transfection breast cancer cells with Arf6-T27N or Arf6 siRNA suppressed EGF-induced *E*-cadherin internalization. Taken together, our study demonstrated that Arf6 activation plays a potential role in EGF-induced *E*-cadherin internalization, providing new mechanism underlying the effect of Arf6 on promoting breast cancer cell metastasis.

**Electronic supplementary material:**

The online version of this article (doi:10.1186/s12935-015-0159-3) contains supplementary material, which is available to authorized users.

## Introduction

Epithelial-mesenchymal transition (EMT) is an essential phenotypic conversion that has been implicated in the initiation of metastasis for breast cancer progression [1,2]. At the invasive front of the breast tumor, EMT is provoked by signals that cells receive from their microenvironment, such as TGF-β, Wnt, and EGF [3-5]. In the subsequent EMT processes, breast cancer cells lose cell-cell junction, and gain migratory and invasive properties, providing them a distinct advantage in tumor progression and metastasis. However, the molecular mechanisms underlying loss of cell-cell junction are poorly understood.

*E*-cadherin is a major component of the adherens junction (AJ), at which it provides cell–cell adhesion through homophilic binding between molecules on adjacent epithelial cells [6]. Previous studies reported that *E*-cadherin loss results in breakage of cell–cell adhesion, and induction of multiple transcription factors, which contributes to EMT and metastatic dissemination [7]. As we know, the situation of *E*-cadherin in epithelial cells is not a static state; it undergoes constitutive internalization and trafficking back to the plasma membrane at the basolateral membrane, and is subject to stringent cellular control [8,9]. Although loss of surface *E*-cadherin has often been linked to repression of *E*-cadherin expression, excessive internalization and/or degradation of *E*-cadherin is also responsible for the down-regulation of surface *E*-cadherin [10]. In fact, *E*-cadherin internalization in response to HGF is accompanied by the disruption of cell-cell adhesion and scattering of cells [11]. Nevertheless, the mechanisms governing *E*-cadherin internalization in breast cancer cells are still need to be explored.

Arf6, a member of the ADP-ribosylation factor (Arf) family, has emerged as a critical regulator of membrane traffic and cytoskeletal organization [12]. Like all GTPases, Arf6 interact with two general types of regulatory proteins: guanine nucleotide exchange factors (GEFs) and GTPase-activating proteins (GAPs). In a study of Mardin-Darby canine kidney (MDCK) epithelial cells, overexpression of SMAP1, a GAP for Arf6, strongly inhibited basal, as well as phorbolester-induced, internalization of *E*-cadherin [13]. By contrast, GEP100, a GEF for Arf6, links EGFR signaling to Arf6 activation to induce invasive activities of breast cancer cells [14]. Since Arf6 has been proposed to function as a critical determinant of disassembly of AJs and cell migration, and loss of functional *E*-cadherin is regarded as a hallmark of EMT and cancer cell invasiveness, thus, it is interesting to explore whether the Arf6 is involved in *E*-cadherin internalization in breast cancer cells.

Recent studies including the results from our laboratory showed that Arf6 activation could be induced by EGF and act as a mediator of cell migration and invasion in various types of cancer including breast cancer cells [14-17]. Here, we used human breast cancer cell lines MCF-7 and T47D to examine the effects of EGF on *E*-cadherin internalization. Using immunofluorescence and immunoblotting analysis, we further explored the involvement of Arf6 in EGF-induced alteration of *E*-cadherin internalization.

## Materials and methods

### Cell culture and transfection

Human breast cancer cell line MCF-7, T47D (ATCC, Manassas, VA) was maintained at 37°C in Dulbecco’s modified Eagle’s medium (DMEM, high glucose) (Hyclone, ThermoScientific, Waltham, MA) supplemented with 10% (v/v) fetal bovine serum (FBS) (Hyclone), 100 U/mL penicillin and 0.1 mg/mL streptomycin in a humidified incubator with 5% CO_2_. Cells were made quiescent by serum starvation overnight followed by treatment with recombinant human EGF (rhEGF, R&D Systems, Minneapolis, MN).

Full-length Arf6-T27N (kindly provided by Dr. Julie G. Donaldson, Laboratory of Cell Biology, NIH) was cloned into pEGFP-N1 vector. Cells, when approximately 80% confluent, were transfected with empty vector or pEGFP-N1 expressing Arf6-T27N using Lipofectamine 2000 as instructed by the manufacturer (Invitrogen, Carlsbad, CA). The sequences of small interfering RNA (siRNA) for Arf6 were as follows: #1, 5′-GUGGCAAAUAAUGAGUAAUTT-3′, #2, 5′-GCGACCACUAUGAUAAUAUTT-3′, and #3, 5′-GACGCCAUAAUCCUCAUCUTT-3′; and the sequence of control siRNA was 5′-UUCUCCGAACGUGUCACGUTT-3′ (GenePharma Co., Shanghai, China). Cells were transfected with control siRNA or Arf6 siRNA with Lipofectamine 2000, according to the manufacturer’s instruction. Cells were allowed to grow for 24 to 48 h post transfection. Before EGF treatment, cells were made quiescent by serum starvation overnight.

### Immunoblotting analysis

Subconfluent cells were washed with PBS, and lysed with RIPA lysis buffer (150 mmol/L NaCl, 50 mmol/L Tris–HCl (pH 7.4), 1% Triton X-100, 1% sodium deoxycholate, 0.1% SDS) with 1 mmol/L sodium orthovanadate, 1 mmol/L PMSF, and 1% cocktail of protease inhibitors (Sigma, St. Louis, MO). The lysates were clarified by centrifugation at 12000 g for 20 min at 4°C and separated by SDS-PAGE followed by transfer onto nitrocellulose membranes. The following antibodies were used: mouse anti-*E*-cadherin antibody (BD Biosciences, San Jose, CA), goat anti-biotin antibody (Sigma), mouse anti-GAPDH antibody (KangChen Bio-tech, Shanghai, China), rabbit anti-GFP antibody (Cell Signaling Technology, Beverly, MA), rabbit anti-Arf6 antibody (Abcam, Cambridge, MA). Protein bands were detected by incubating with horseradish peroxidase-conjugated secondary antibodies (Santa Cruz Biotechnology) and visualized with ECL reagent (Millipore). Digital images of immunoblots were obtained with a Chemidoc XRS and analyzed using the image analysis program Quantity One (Bio-Rad, Hercules, CA).

### Immunofluorescence microscopy

Cells adhered on glass cover slips were fixed with 4% paraformaldehyde for 20 min, washed with PBS, and then permeabilized in 0.1% Triton X-100/PBS. After blocking in PBS containing 1% bovine serum albumin (BSA) for 1 h at room temperature, the cells were incubated with primary antibody overnight at 4°C followed by incubation with FITC or rhodamine conjugated secondary antibody for 1 h at room temperature within a moist chamber. Following wash with PBS, the cover slips were mounted on glass slides with DAPI Fluoromount G (Southern Biotech, Birmingham, AL). Images were acquired with an Olympus BX51 microscope coupled with an Olympus DP70 and prepared for publication with Adobe Photoshop (Adobe Systems, Unterschleissheim, Germany).

### Pulldown assays

Active Arf6 was measured as instructed by the manufacturer (Promega, Madison, WI). GST-GGA3 (a gift from Drs. James E. Casanova and Kathryn Davis, University of Virginia, VA) was used for capturing active Arf6 in cell lysates. Briefly, the GST fusion proteins were purified from BL21 bacteria and isolated by incubation with MagneGST Glutathione Particles (Promega) for 30 min at 4°C. After treatment of cells with the appropriate stimuli, cells were lysed and equal volumes of total cellular protein were incubated with particles carrying GST-fusion protein for 1 h on a rotating wheel at 4°C. The particles were then washed five times with Binding/Wash Buffer (4.2 mmol/L Na_2_HPO_4_, 2 mmol/L KH_2_PO_4_, 280 mmol/L NaCl, and 10 mmol/L KCl, pH 7.2), solubilized in 1 × SDS sample buffer and then subjected to SDS-PAGE and immunoblotted with antibody against Arf6.

### Internalization assay

Quantification of internalized biotinylated *E*-cadherin at the cell surface was carried out by international assay. Briefly, the cell surface was labeled for 1 h on ice with 0.2 mg/mL cleavable, membrane-impermeable EZ Link Sulfo-NHS-SS-Biotin (Thermo Fisher Scientific Inc, Rockford, IL) in PBS supplemented with 1 mmol/L CaCl_2_ and 1 mmol/L MgCl_2_. After quenching with DMEM, one sample of the cells was directly lysed and the remaining samples were incubated in DMEM with or without EGF at 37°C for the indicated periods. Subsequently, surface biotin was stripped by two 20 min washes of glutathione solution (50 mmol/L glutathione, 75 mmol/L NaCl, 75 mmol/L NaOH, and 1% BSA) at 0°C. Remaining biotinylated proteins were sequestered inside cells by endocytosis and were therefore protected from glutathione stripping. Cells were then washed, lysed and incubated with streptavidin beads (Sigma) overnight at 4°C to capture the biotinylated proteins, and then the samples were resolved by SDS-PAGE and immunoblotted with antibody against *E*-cadherin.

### Co-immunoprecipitation

Cells were lysed in lysis buffer as described above and 200 μg fresh protein was incubated with anti-*E*-cadherin antibody at 4°C overnight followed by 2 h incubation with Protein A + G Agarose (Beyotime, Nantong, China). The beads were washed three times, solubilized in 1 × SDS sample buffer and resolved by SDS-PAGE followed by immunoblotting analysis.

### Statistical analysis

Data were analyzed by ImageJ and statistical analyses were carried out using the SPSS software version 15.0 (SPSS Inc., Chicago, IL). Student’s *t* test was used to analyze differences between two groups. Statistical significance was considered when *P* < 0.05.

## Results

### EGF induces internalization of E-cadherin in breast cancer cells

**Figure 1:**
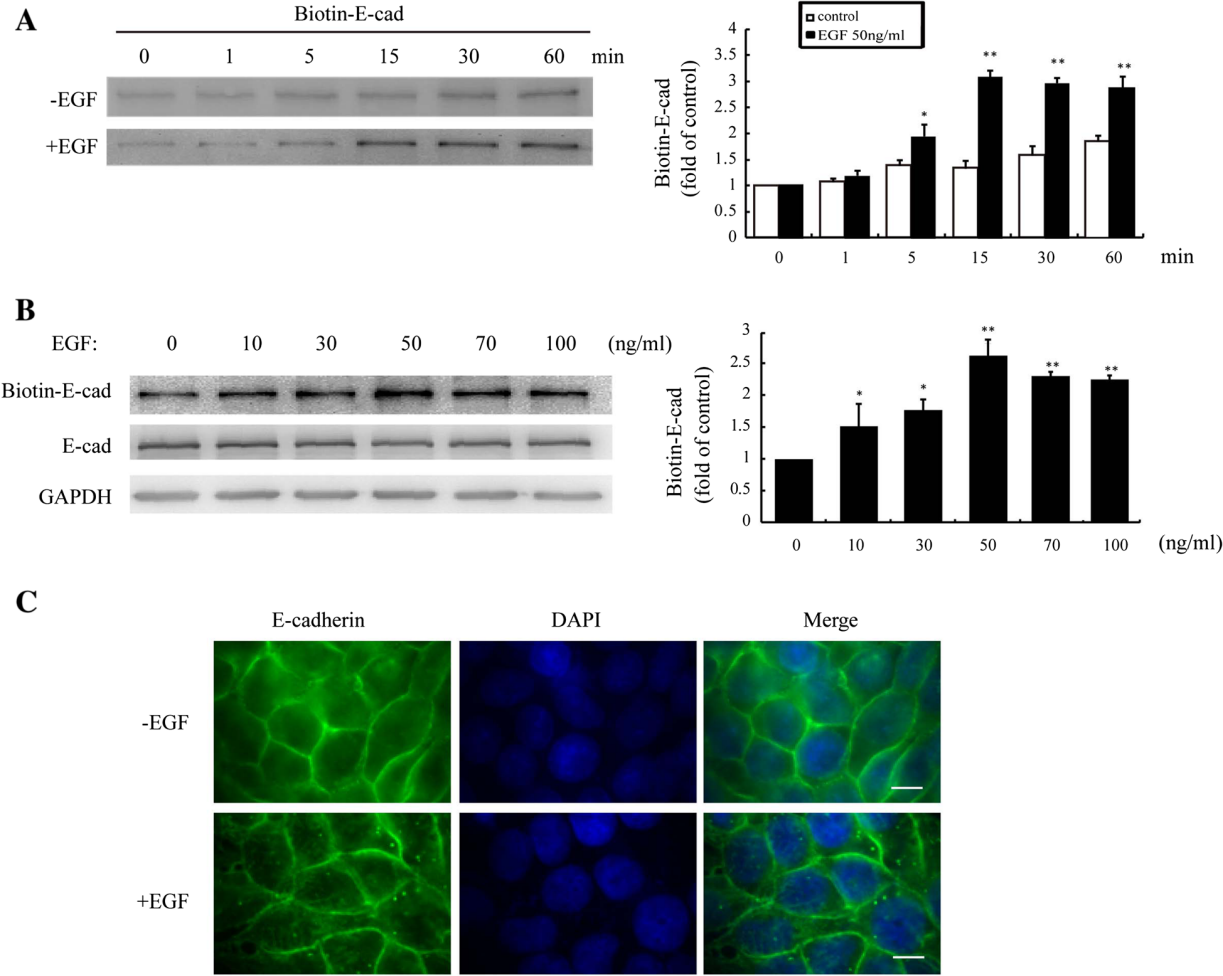
**EGF promotes internalization of** ***E***-**cadherin in MCF**-**7 cells. (A)** Levels of biotin-labeled *E*-cadherin in MCF-7 cells under EGF (50 ng/mL) treatment for up to 60 min were analyzed by internalization assays. **(B)** Levels of biotin-labeled *E*-cadherin under different concentrations of EGF for 15 min were analyzed by internalization assays. **(C)** Representative micrographs of cells treated with EGF (50 ng/mL) for 15 min and stained for distribution of *E*-cadherin using FITC-conjugated secondary antibody (green). Cells were counterstained with DAPI (blue). Images are representative of at least 3 independent determinations. Scale bar, 10 μm. *: *P* < 0.05, **: *P* < 0.01, referring to the difference between cells treated with and those without EGF.

To assess the effect of EGF on *E*-cadherin internalization in breast cancer cells, we treated MCF-7 cells with EGF, and measured internalized *E*-cadherin levels by internalization assays. As described in Materials and Methods, surface proteins of MCF-7 cells were labeled with cleavable biotin. Compared with control cells, a greater amount of biotinylated *E*-cadherin accumulated in the cytoplasm of MCF-7 cells treated with EGF for 15 min (Figure 1A). To determine the optimal concentration of EGF for *E*-cadherin internalization, we allowed surface-biotinylated *E*-cadherin to be internalized over a range of EGF concentrations. We found that EGF potently stimulated *E*-cadherin internalization, which peaked at 50 ng/ml EGF, with an approximately 2-fold increase over untreated cells (Figure 1B). Of note, the total amount of *E*-cadherin remained unchanged after EGF stimulation in all cells (Figure 1B). Furthermore, immunofluorescence assays revealed a significantly greater number of internalized *E*-cadherin particles in MCF-7 cells following incubation with 50 ng/mL EGF than that of untreated cells (Figure 1C). Accordingly, EGF was used at 50 ng/mL in subsequent experiments.

### E-cadherin forms complexes with Arf6

**Figure 2:**
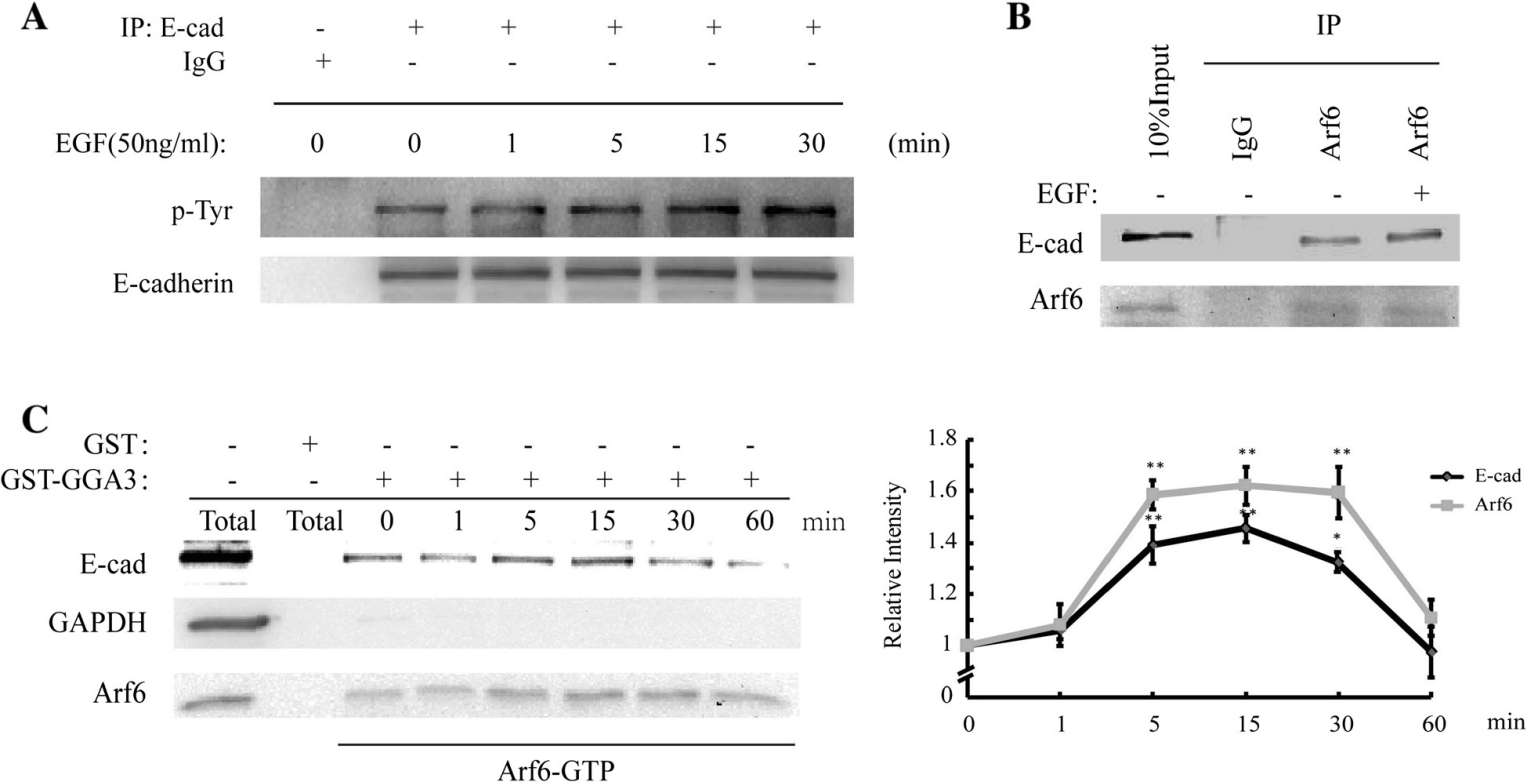
**EGF stimulates interaction of** ***E***-**cadherin with Arf6. (A)** Effect of EGF on the tyrosine phosphorylation of *E*-cadherin under EGF (50 ng/mL) treatment for up to 30 min were determined by immunoprecipitation; GAPDH was used as a loading control. n = 3 for the experiments above. **(B)** Cells treated with EGF (50 ng/mL, 15 min) were immunoprecipitated with anti-Arf6 antibody, followed by immunoblotting for *E*-cadherin. The second top panel shows immunoprecipitated Arf6. **(C)** Co-immunoprecipitation of *E*-cadherin by activated Arf6 was determined. Expression of activated Arf6 in total cell lysates is shown in the bottom panels. n = 3 for all experiments. *: *P* < 0.05, **: *P* < 0.01, referring to the difference between cells treated with and those without EGF.

Although Arf6 was proved to be involved in EGF-induced *E*-cadherin internalization, the precise mechanisms underlying this regulation were poorly known. We therefore we sought to examine whether tyrosine phosphorylation of *E*-cadherin occurred in our experimental system. MCF-7 cells treated with EGF at different time points were lysed, and followed by immunoprecipitation and immunoblotting as indicated. We found that *E*-cadherin only showed visible signs of basal tyrosine phosphorylation, which was elevated after stimulation with EGF and peaked at 15-30 min (Figure 2A). Next, we analyzed whether a physical interaction between Arf6 and *E*-cadherin existed in MCF-7 cells. We examined the physical interaction between these two proteins in MCF-7 cell lysates by co-immunoprecipitation assay using an anti-Arf6 antibody. We observed that the association between Arf6 and *E*-cadherin was significantly increased in EGF-treated cells than that in untreated cells (Figure 2B). To determine if this association was related with Arf6 activation, we turned to pulldown assay. Likewise, activated Arf6 was found to associate with *E*-cadherin, and *E*-cadherin was pulled down in greater amounts with Arf6 when the activity of the latter was increased (Figure 2C). These results showed that *E*-cadherin became associated with Arf6 during internalization, which may profit from the activation of Arf6.

### EGF-induced E-cadherin internalization requires Arf6 activation

**Figure 3:**
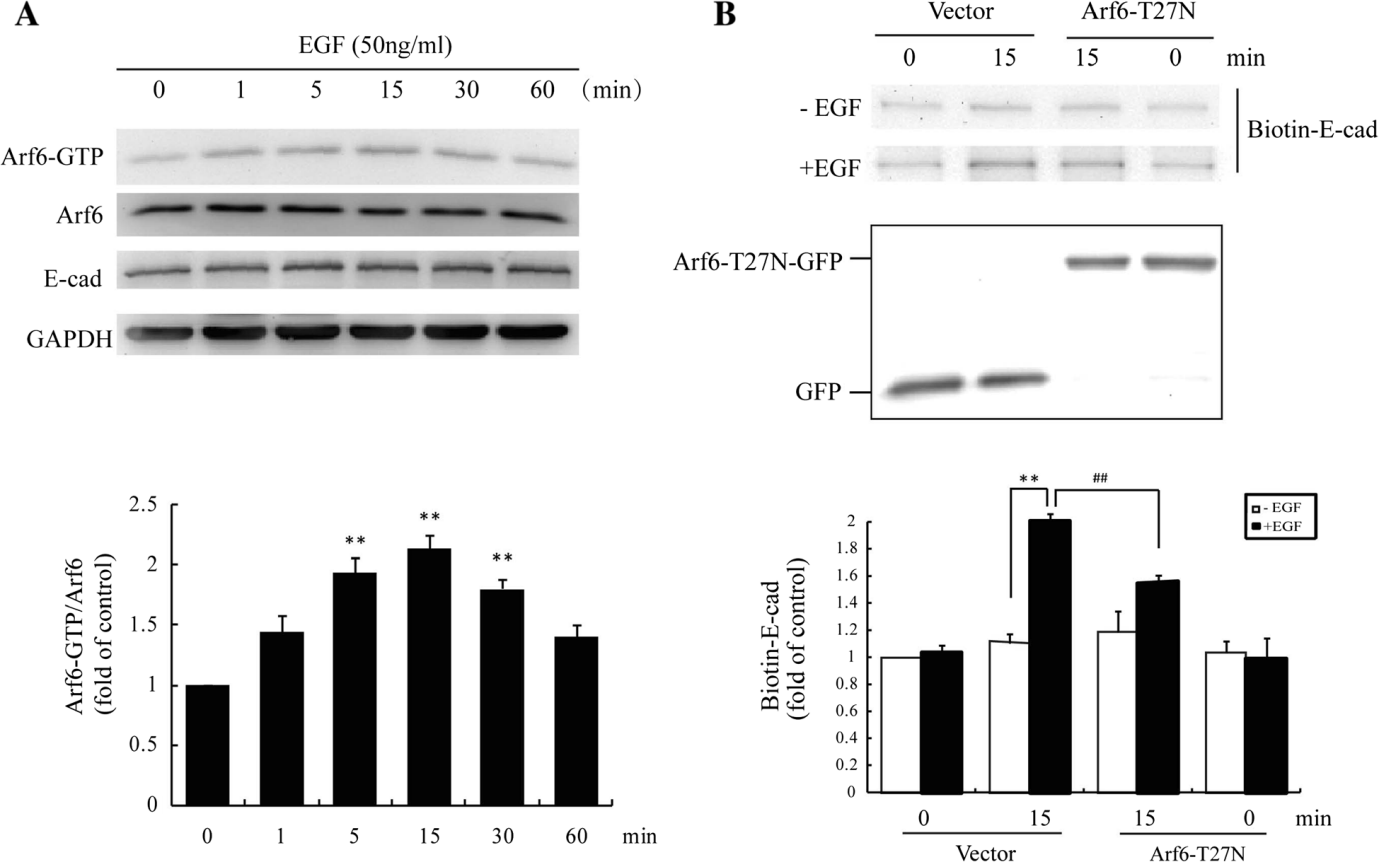
**Arf6 activation is required for internalization of** ***E***-**cadherin by EGF. (A)** MCF-7 cells were treated with EGF (50 ng/mL) for up to 60 min, and Arf6 activation were determined by pulldown assay. **: *P* < 0.01, referring to the difference between cells treated with and those without EGF. **(B)** Expression of biotin-labeled *E*-cadherin was determined by internalization assays in the MCF-7 cells transiently transfected with empty vector or Arf6-T27N. Representative Western blots from 3 independent experiments are shown. **: *P* < 0.01, referring to the difference between cells treated with and those without EGF. ^##^: *P* < 0.01, referring to the difference between cells transfected with Arf6–T27N plus EGF and the cells transfected with empty vector plus EGF.

**Figure 4:**
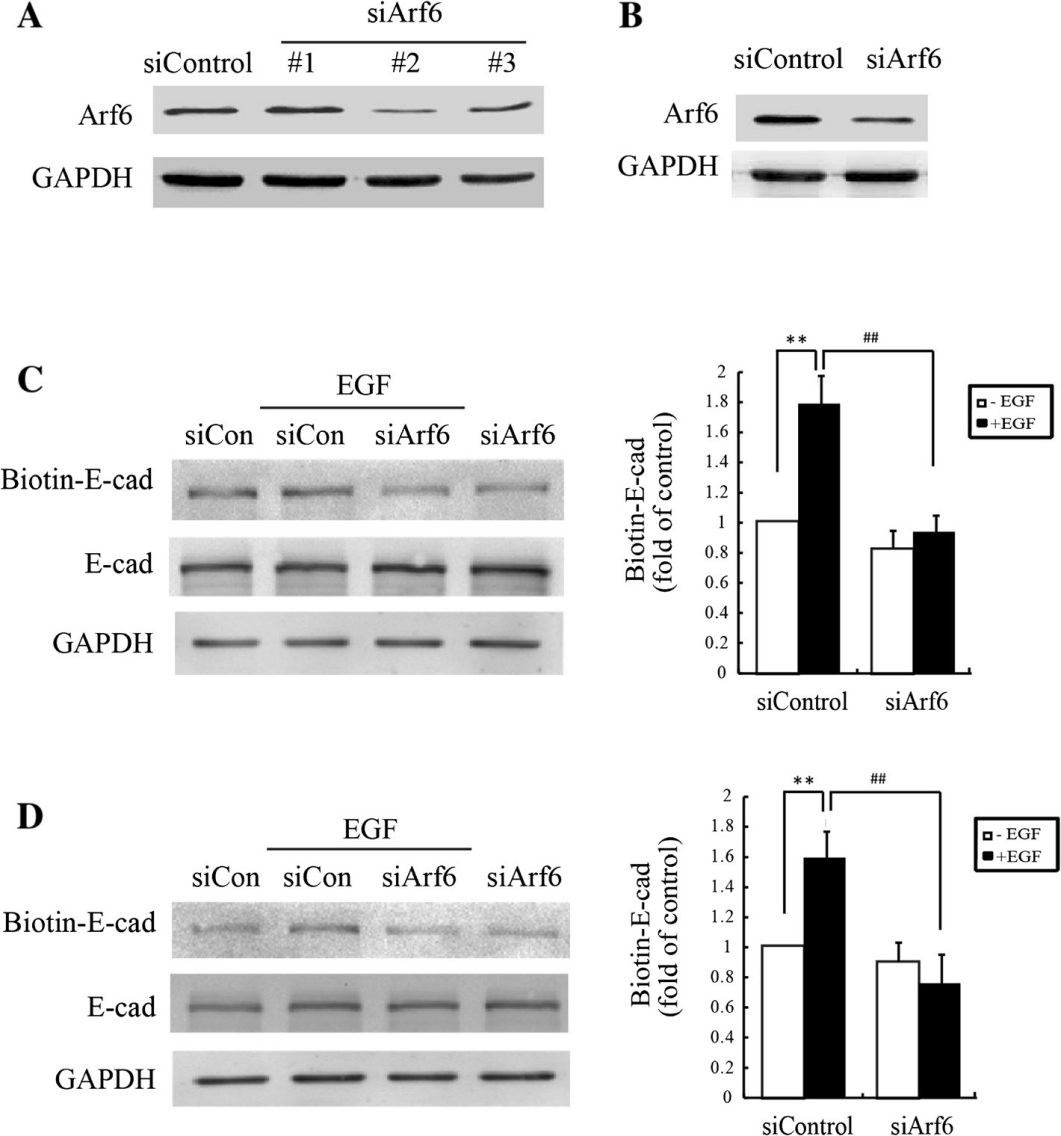
**Silencing Arf6 reduces internalization of** ***E*** **-cadherin by EGF. (A&B)** The expression of Arf6 after treatment with Arf6 siRNA. Total protein extracts from T47D **(A)** and MCF-7 **(B)** cells transfected with Arf6 siRNA or control siRNA (mock) were analyzed by immunoblotting for Arf6. GAPDH was used a loading control. **(C&D)** Expression of biotin-labeled *E*-cadherin was determined by internalization assays in the T47D **(C)** and MCF-7 **(D)** cells transfected with siRNA for Arf6. **: *P* < 0.01, referring to the difference between cells treated with and those without EGF. ^##^: *P* < 0.01, referring to the difference between cells transfected with Arf6 siRNA plus EGF and the cells transfected with control siRNA plus EGF.

**Figure 5:**
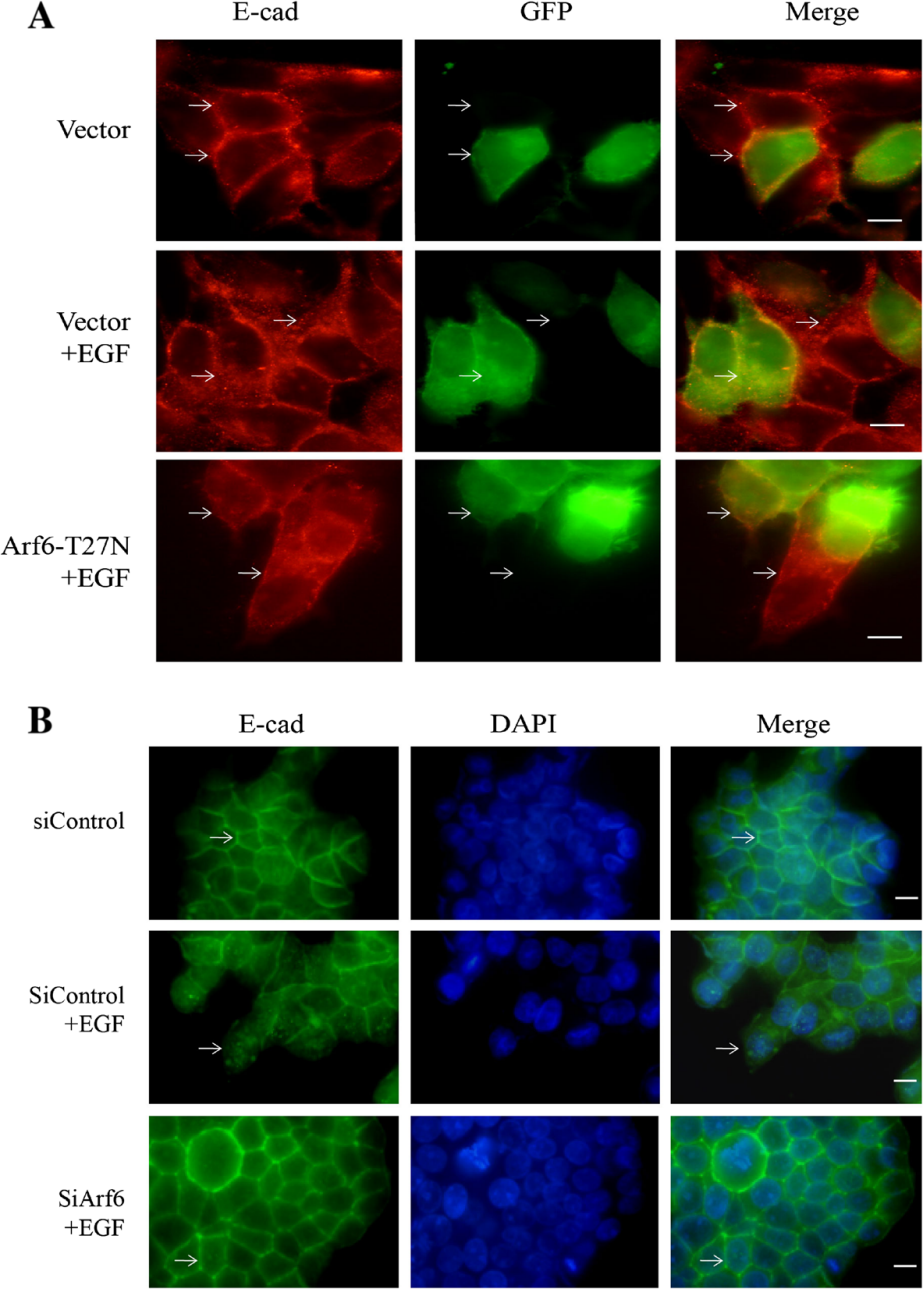
**Effect of Arf6 inactivation on** ***E*** **-cadherin location in breast cancer cells. (A)** Representative micrographs of MCF-7 cells treated with EGF and stained for *E*-cadherin. Cells transfected with Arf6-T27N or empty vector (GFP, green) were grown under EGF (50 ng/mL) for 15 min and examined *E*-cadherin expression (red) by immunofluorescence assay. The arrow shows that cells transfected with GFP-Arf6-T27N showed weaker *E*-cadherin internalization than control cells (red) after EGF treatment. **(B)** Representative micrographs of T47D cells treated with EGF and stained for *E*-cadherin. Cells transfected with Arf6 siRNA were grown under EGF (50 ng/mL) for 15 min and examined *E*-cadherin expression (green) by immunofluorescence assay. The arrow shows that cells transfected with Arf6 siRNA showed weaker *E*-cadherin internalization than control cells after EGF treatment. Scale bar, 10 μm. Images are representative of at least 3 independent determinations.

We next examined whether endogenous Arf6 activation also changed after EGF treatment by pulldown assays. We observed that Arf6-GTP was significantly elevated at 5 min after stimulation with 50 ng/mL EGF with maximal activation at 15 min (Figure 3A), suggesting that Arf6 may participate in the regulation of *E*-cadherin internalization. To confirm this supposition, we transfected MCF-7 cells with GFP-tagged Arf6-T27N plasmid, a dominant negative construct of Arf6. The internalization assays showed that internalized biotinylated *E*-cadherin by EGF was much less in Arf6-T27N transfected cells than those transfected with empty GFP vectors (Figure 3B). As shown in Fig4A&4B, Arf6 siRNA (#2) greatly knocked down Arf6 expression in T47D (Figure 4A) and MCF-7 cells (Figure 4B), as assessed by immunoblotting analysis. Accordingly, Arf6 siRNA (#2) was used in subsequent experiments. As expected, Arf6 knockdown resulted in a significant reduction of EGF-induced E-cadherin internalization in both T47D (Figure 4C) and MCF-7 cells (Figure 4D). The results were confirmed by immunofluorescence assays showing that EGF-induced *E*-cadherin internalization was largely abolished by transfection with Arf6-T27N plasmid in MCF-7 cells (Figure 5A). In T47D cells, EGF-induced *E*-cadherin internalization was also suppressed by transfection with Arf6 siRNA (Figure 5B). Taken together, these results demonstrated that Arf6 activation is required for EGF-induced *E*-cadherin internalization in breast cancer cells.

## Discussion

EGF signaling is implicated in regulating mammary gland morphogenesis and development, while aberrant EGFR activity is associated with EMT-associated migration and invasion in normal and malignant mammary epithelial cells [5]. The focus of this study was to determine the mechanism through which EGF regulates *E*-cadherin distribution in breast cancer cells, which is regarded as the first step of EMT. Our results indicate that Arf6 plays an important role in the regulation of *E*-cadherin internalization in response to EGF, and suggest that Arf6 may exert its function by physically interacting with *E*-cadherin in breast cancer.

A primary observation in the present study is that EGF enhances internalization of *E*-cadherin from cell membranes without affecting the total protein level of *E*-cadherin, suggesting that *E*-cadherin is internalized but not degraded in our observation period. In parallel, enhanced co-localization between *E*-cadherin and early endosome antigen 1 (EEA1) was observed (Additional file 1: Figure S1). EEA1 has an important role in endosomal trafficking and is localized exclusively to early endosomes. The results suggest that *E*-cadherin is localized to endosomes after EGF stimulation and then may be targeted to either the recycling or lysosome-dependent degradation pathway.

An association between *E*-cadherin phosphorylation and its internalization has been reported. In some cell types, *E*-cadherin is known to be highly phosphorylated within the Ser cluster in the cytoplasmic domain [18]. Interestingly, we observed that EGF stimulation was accompanied by increased tyrosine phosphorylation of *E*-cadherin. Our result is confirmed by the demonstration that point mutation of tyrosine phosphorylated site of vascular *E*-cadherin prevents vascular *E*-cadherin internalization in response to bradykinin [19]. Although phosphorylation of *E*-cadherin by PKD1 is reportedly associated with increased cellular aggregation and decreased cellular motility in prostate cancer [20], our result is consistent with the demonstration that *E*-cadherin tyrosine-phosphorylation status contributes to its ubiquitination and subsequent increase in cell migration [21]. Therefore, it may be reasonable to think that in MCF-7 breast cancer cells, EGF-induced *E*-cadherin internalization could be mediated by its tyrosine phosphorylation modifications.

Arf6 can be activated by various growth factors, such as vascular growth factor [22], colony-stimulating factor [23], and G protein coupled receptor agonists [24]. Recent studies including the results from our laboratory showed that EGF treatment also could induce Arf6 activation and increased breast cancer cell migratory potential [14-16]. It should be mentioned that several studies have examined the role of Arf6 function in *E*-cadherin trafficking, but controversy still remains. Palacios *et al*. reported that the expression of Arf6-Q67L, a dominant positive construct of Arf6, induced a loss of *E*-cadherin from AJs in MDCK cells [25]. In HepG2 cells, depletion of GEP100, one special GEF for Arf6, resulted in upregulation of *E*-cadherin content and blockade of *E*-cadherin redistribution induced by HGF [26]. Conversely, Paterson *et al*. found that expression of Arf6-Q67L prevented internalization of *E*-cadherin into diffuse small vesicles, while Arf6-T27N expression had no apparent effect on *E*-cadherin internalization [27]. We show here that EGF triggers a rapid stimulation of Arf6 activity. When Arf6 activity was blocked by ectopic expression of a dominant-negative Arf6 mutant, or silenced by Arf6 siRNA, EGF-stimulated *E*-cadherin internalization was dramatically diminished. Therefore, our results suggest that Arf6 activation serves as a mediator of EGF-stimulated *E*-cadherin internalization in breast cancer cell. The different results gained by different groups may be due to the different cell systems used and receptor-coupled status in these studies.

*E*-cadherin can form multicomponent complexes with EGFR and other receptor tyrosine kinases (RTKs) at the basolateral areas of polarized epithelial cells [28-30]. Here, we noticed that Arf6 binds to *E*-cadherin in MCF-7 cells. Bach *et al*. have announced that *M*-cadherin recruited a multi-protein “fusion complex” composed of Arf6, Trio, and Rac1 in C2C12 mouse myoblasts [31]. As members of the cadherin superfamily, *E*- and *M*-cadherin share similar structures, and it is not surprising that *E*-cadherin can associate with Arf6. Our results also showed that co-localization between Arf6 and *E*-cadherin became stronger in EGF-stimulated cells. In addition, *E*-cadherin became associated with more Arf6 with increased activation of Arf6, which decreased when Arf6 was inactivated. Therefore, it is reasonable to think that Arf6 may promote *E*-cadherin internalization through physical association with *E*-cadherin on its activated state, although the domain in Arf6 that binds to *E*-cadherin needs to be further investigated.

In summary, this study highlights the role of Arf6 that accounts for *E*-cadherin internalization. Arf6 may function on its GTP-bounded status to promote EGF-stimulated *E*-cadherin internalization in breast cancer cells. These findings are of potential pathophysiological importance for understanding Arf6 which mechanistically behaves as a tumor promoter that leads to structural loss of adhesion and contributes to aggressive phenotypes in breast cancer.

## Additional file

Additional file 1: Figure S1. Internalized *E*-cadherin enters the early endosomal compartment. Representative micrographs of cells treated with EGF (50 ng/mL) for 15 and 60 min and stained for distribution of *E*-cadherin using FITC-conjugated secondary antibody (green) and EEA1 using rhodamine-conjugated secondary antibody (red). Scale bar, 10 μm.
