# Supplementary figures and images for: Arf6 regulates EGF-induced internalization of E-cadherin in breast cancer cells
Source: Cancer Cell Int. 2015 Feb 4;15(1):11. doi: 10.1186/s12935-015-0159-3 (PMC4326200; doi:10.1186/s12935-015-0159-3)

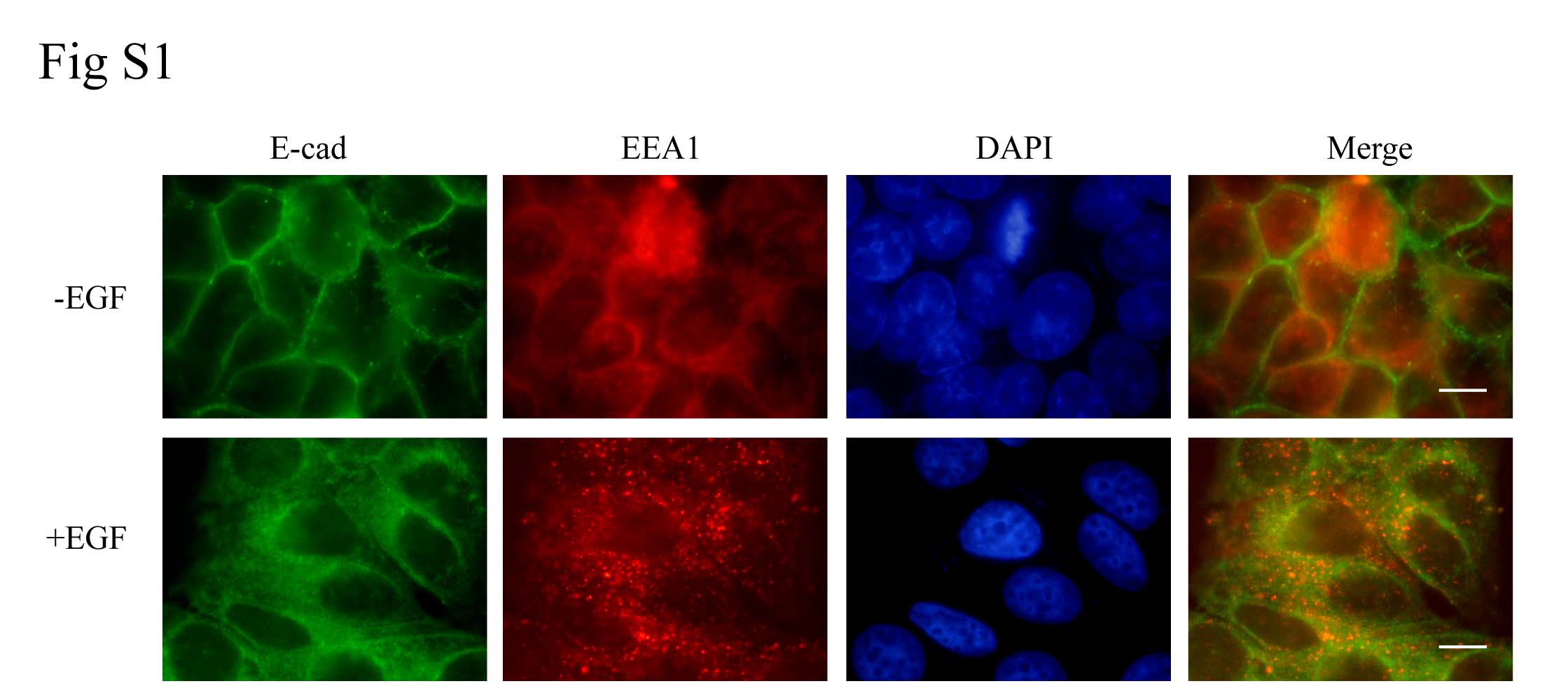

Supplement: Additional file 1: Figure S1. — Internalized E-cadherin enters the early endosomal compartment. Representative micrographs of cells treated with EGF (50 ng/mL) for 15 and 60 min and stained for distribution of E-cadherin using FITC-conjugated secondary antibody (green) and EEA1 using rhodamine-conjugated secondary antibody (red). Scale bar, 10 μm. [file 12935_2015_159_MOESM1_ESM.jpeg]
